# HTLV-1/2 in Indigenous Peoples of the Brazilian Amazon: Seroprevalence, Molecular Characterization and Sociobehavioral Factors Related to Risk of Infection

**DOI:** 10.3390/v15010022

**Published:** 2022-12-21

**Authors:** Isabella Nogueira Abreu, Carlos Neandro Cordeiro Lima, Eliene Rodrigues Putira Sacuena, Felipe Teixeira Lopes, Maria Karoliny da Silva Torres, Bernardo Cintra dos Santos, Vanessa de Oliveira Freitas, Leonardo Gabriel Campelo Pinto de Figueiredo, Keise Adrielle Santos Pereira, Aline Cecy Rocha de Lima, Wandrey Roberto dos Santos Brito, Bruno José Sarmento Botelho, Janete Silvana Souza Gonçalves, Sandra Souza Lima, Izaura Maria Vieira Cayres Vallinoto, João Farias Guerreiro, Ricardo Ishak, Antonio Carlos Rosário Vallinoto

**Affiliations:** 1 Laboratório de Virologia, Universidade Federal do Pará, Belém 66075-110, Brazil; 2 Programa de Pós-Graduação em Biologia de Agentes Infecciosos e Parasitários, Instituto de Ciências Biológicas, Universidade Federal do Pará, Belém 66075-110, Brazil; 3 Laboratório de Genética Humana e Médica, Universidade Federal do Pará, Belém 66075-110, Brazil

**Keywords:** HTLV-1/2, epidemiology, Indigenous people, Brazilian Amazon

## Abstract

HTLV-1/2 infection is endemic in Indigenous peoples of the Americas. Its origin is attributed to the migratory flow of Amerindian ancestral peoples. The present study aimed to investigate the seroprevalence of HTLV-1/2 infection in Indigenous peoples of the Brazilian Amazon. A total of 3350 Indigenous people belonging to 15 communities were investigated. The investigation was performed using serological (ELISA), molecular (qPCR) and confirmatory (Western blot and/or Inno-Lia) tests to detect and differentiate the infection. The seroprevalence was 8.3% for HTLV-1/2 infection, with 0.1% of individuals seropositive for HTLV-1 and 8.1% for HTLV-2. The prevalence of infection was statistically higher in women (10.1%) than in men (6.5%) (*p* = 0.0002). This female predominance was observed in all age groups; in females the prevalence was significant from 41 years old (*p* < 0.0001) and in males from 51 years old (*p* < 0.0001). Here, we present a prevalence of HTLV-1/2 among Indigenous peoples of the Brazilian Amazon. The endemic infection in these groups must reflect the different epidemiological profiles observed in these peoples, such as sexual transmission through rejection of condom use, breastfeeding, especially in cases of cross-breastfeeding, and the high rate of pregnancy in the villages.

## 1. Introduction

Human T-lymphotropic viruses 1 and 2 (HTLV-1 and HTLV-2) are grouped in the *Retroviridae* family and have tropism for CD4+ and CD8+ T cells, respectively [1,2].

It is estimated that 5 to 10 million people worldwide are infected with HTLV-1, with the geographical distribution of the virus being heterogeneous, with some regions considered endemic, such as Japan, Africa, the Caribbean, North America, South America, the Middle East and the Australo-Melanesia region [3,4,5]. HTLV-2, in turn, is predominantly found in drug users and Indigenous populations, with the Americas, Europe and Central Africa as endemic regions for the infection. The Indigenous populations of the Brazilian Amazon have already been described as endemic for HTLV-2, especially among the Kayapó ethnic group (Gorotire, Kararaô, Xikrin do Bacajá, Kubenkokre and Kokraimoro subgroups), as well as the Munduruku, Parakanã and Arara do Laranjal peoples [6,7,8].

It is suggested that the origin of HTLV occurred on the African continent through interspecies transmission from the infection of humans with simian T-lymphotropic viruses 1 and 2 (STLV-1/2). The spread of the virus occurred approximately 100,000 years ago, with the departure of man from Africa toward Europe, Asia and the Americas through the Bering Strait [9,10,11].

Among the HTLV-1/2 transmission routes, mother-to-child transmission is important for the maintenance and endemicity of the virus, particularly HTLV-2 among Indigenous peoples, which is commonly associated with prolonged breastfeeding and cross-breastfeeding (when a woman breastfeeds a child who is not her child). The sexual route is also important, since condom use is uncommon in these people, and the infection is more likely to be transmitted from men to women [12,13,14].

To date, only HTLV-1 infection has been associated with inflammation, malignancy and neurological diseases, affecting approximately 5% of people living with HTLV-1 and being able to trigger adult T-cell leukemia/lymphoma (ATL), HTLV-1-associated myelopathy (HAM) and other diseases, such as uveitis, dermatitis, alveolitis, arthritis, Sjogren’s syndrome, cystitis, prostatitis and polymyositis [15,16,17,18]. HTLV-2 infection is usually asymptomatic, with rare cases associated with neurological diseases resembling HAM [19,20,21,22,23,24].

The present study describes the prevalence of HTLV-1/2 infection in 15 Indigenous peoples from the Brazilian Amazon (six communities visited for the first time), the molecular characterization of the virus and the sociobehavioral aspects of risk for infection.

## 2. Materials and Methods

### 2.1. Study Population

From June 2018 to March 2022, health actions were carried out in 10 municipalities in the State of Pará, aiming at the clinical and laboratory evaluation of 15 Indigenous communities, with blood samples being collected to investigate HTLV-1/2 infection. The study was carried out by the Laboratory of Virology and the Laboratory of Medical and Human Genetics of the Federal University of Pará, with the support of state government agencies (State Coordination of Indigenous Health and Traditional Peoples—CESIPT, the Integrated Center for Inclusion and Rehabilitation—CIIR, the Health Secretariat of Pará—SESPA and the Special Secretariat for Social Communication—SECOM, together with the Special Indigenous Health Districts—DSEIs, the Special Secretariat for Indigenous Health—SESAI, the Ministry of Health, and those responsible for indigenous health in Brazil).

**Figure 1:**
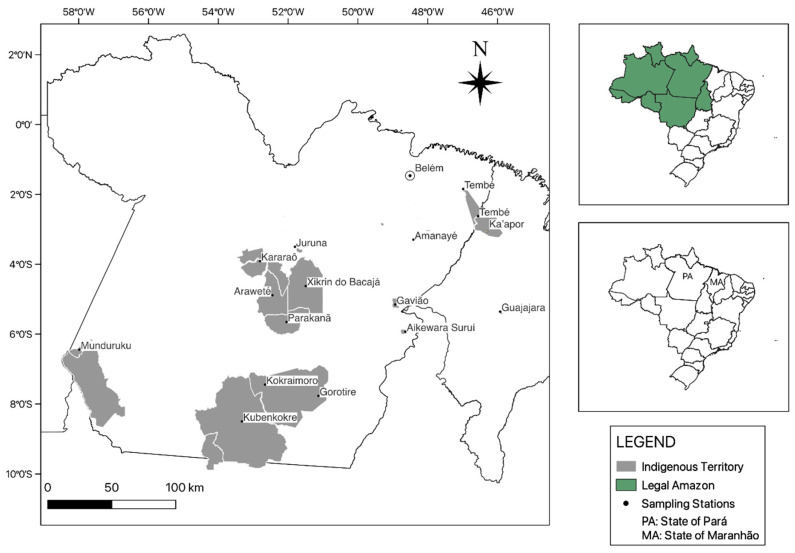
Geographic location of the studied Indigenous territories.

Indigenous people of both sexes and all age groups participated in the study (Table 1), including groups with different degrees of multiraciality and contact with nonindigenous populations: Aikewara-Suruí, Araweté, Amanayé, Munduruku, Parakanã, Tembé, Gavião, Juruna and the Kayapó, from the Xikrin do Bacajá, Kubenkokre, Gorotire, Kokraimoro and Kararaô communities, all located in the State of Pará (Table 2 and Figure 1). Indigenous people from the Ka’apor and Guajajara peoples, residents of the State of Maranhão, were included.

### 2.2. Sample Collection

Venous blood samples (4 mL) were obtained from 3350 Indigenous people using a vacuum collection system in tubes containing EDTA (ethylenediaminetetraacetic acid) as an anticoagulant. Separation of samples into plasma and leukocytes was performed by centrifugation (4000 rpm for 15 min), followed by storage in cryogenic tubes at −80 °C until processing at the Virology Laboratory (LABVIR).

Of the total number of individuals sampled, 1240 agreed to answer a questionnaire with questions about behavioral risk factors for exposure to HTLV-1/2 (“piercing”, “blood transfusion”, “breastfeeding as a child”, “sexual activity”, “condom use”, “number of sexual partners”, “first sexual intercourse”, “if woman, how many pregnancies, duration of breastfeeding and if she has already breastfed other children in the village”).

### 2.3. Serological Analysis

To detect total anti-HTLV-1/2 antibodies, plasma samples were tested in duplicate using an enzyme-linked immunosorbent assay (ELISA) (MUREX HTLV-I + II, DiaSorin, Dartford, UK) following the protocol from the manufacturer.

### 2.4. Molecular Analysis

Samples with positive ELISA results were subjected to DNA extraction using the QIAamp DNA mini kit (Qiagen, Hilden, Germany), followed by a real-time PCR (qPCR) reaction, with the aim of confirming the infection and differentiating the viral type from the amplification of the HTLV-1 pol (186 bp) and HTLV-2 tax (75 bp) genes. Amplification of the human albumin gene (141 bp) was used as an endogenous control [25]. For each reaction, 12.5 μL of TaqMan Universal PCR Master Mix (2×), 6.0 μL of water (H_2_O), 0.5 μL of each primer, 0.5 μL of each probe and 5.0 μL of DNA resulted in a total volume of 25 μL. The following temperature cycles were used: 95 °C for 10 min, followed by 45 cycles of 95 °C for 15 s and 60 °C for binding of primers and probes for 1 min. The primers used were 5′-CCCTACAATCCAACCAGCTCAG-3′ (HTLV-1F), 5′-GTGGTGAAGCTGCCATCGGGTTTT-3′ (HTLV-1R), 5′-CGATTGTGTACAGGCCGATTG-3′ (HTLV-2F), 5′-CAGGAGGGCATGTCGATGTAG-3′ (HTLV-2R), 5′-GCTGTCATCTCTCTTGTGGGCTGT-3′ (Albumin F), 5′-AAACTCATGGGAGCTGCTGGTT-3′ (Albumin R), and probe sequences FAM-5′-CTTTACTGACAAACCCGACCTACCCATGGA-3′-NFQ (HTLV-1), FAM-5′-TGTCCCGTCTCAGGTCTATGTTCCA-3′-NFQ (HLTV-2) and FAM-5′-CCTGTCATGCCCACACAAATCTC-3′-NFQ (Albumin) [25].

### 2.5. Complementary Confirmatory Analyses

Samples from which it was not possible to obtain leukocytes for DNA extraction and those negative in qPCR, in order to rule out possible cases of false negatives, were submitted to the complementary tests using in-line immunoassay INNO-LIA HTLV-I/II Score (Fujirebio, Tokyo, Japan) and enzyme immunoassay HTLV Blot 2.4 kit (MP Diagnostics, Singapore, Republic of Singapore).

### 2.6. Statistical Analysis

The characteristics of the population and the demographic data obtained from the questionnaires were described using descriptive statistical analyses. To identify risk associations for HTLV-1/2 infection, Pearson’s chi-square test and G test were applied considering a significance level of 5% (*p* value < 0.05). All analyses were performed using BioEstat version 5.0.

## 3. Results

### 3.1. Serological and Molecular Analyses

Of the 3350 samples tested, 8.6% (289) were positive in the ELISA test. Among the seropositive samples, 282 were confirmed using qPCR, while the others (*n* = 7), in the absence of leukocyte samples for DNA extraction, were confirmed by Inno-Lia/Western blot (Table 2).

**Figure 2:**
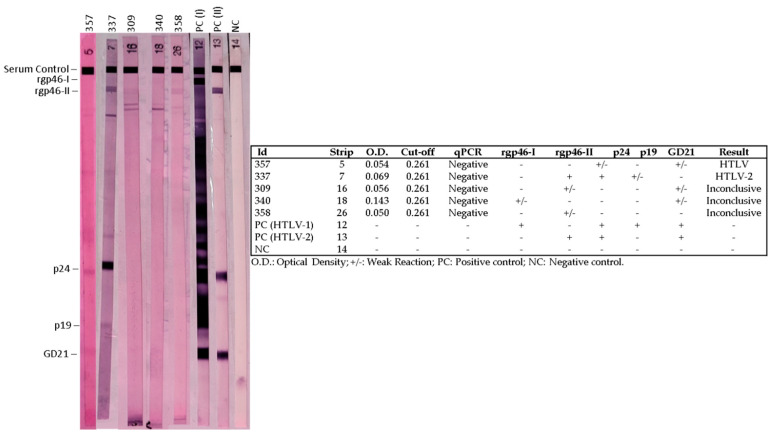
HTLV-1/2 reactivity patterns on Western blot analysis. Strips 5, 7, 16, 18 and 26 correspond to samples from the Kayapó people, 4 from the Kararaô subgroup (strips 7, 16, 18 and 26) and one from the Xikrin do Bacajá subgroup (strip 5). Strips 12, 13 and 14 correspond to the HTLV-1 positive, HTLV-2 and negative controls, respectively.

Of the 40 individuals residing in the Kararaô community, all showed negative results on ELISA; however, considering previous results of the occurrence of HTLV-2 infection in this ethnic group, the samples were analyzed using qPCR and Western blot (Figure 2), which revealed reactivity of one sample with the HTLV-2 pattern (reactivity for rgp46-II, p24 and p19), one HTLV (reactivity for p24 and GD21) and three with inconclusive band patterns (no correspondence to those described by the manufacturer), while in qPCR no sample was reactive. One of these individuals (#357) was of Kaypó origin (Xikrin subgroup of Bacajá) who, after interethnic marriage, currently resides with his Kararaô family.

Of the samples tested using qPCR, 1.2% (4/322) had confirmed HTLV-1 infection and 76.1% (245/322) had HTLV-2, while 22.7% (73/322) had negative results. Of the 73 samples that were negative on the molecular biology test and the seven that had plasma only, 41 were tested using Western blot and 39 using INNO-LIA. HTLV-2 was found in 36.2% (29/80), 6.2% (5/80) were HTLV positive, 10.0% (8/80) were undetermined and 47.5% (38/80) were negative (Table 2).

The overall prevalence of HTLV-1 infection was 0.1% (4/3350) and 8.2% (274/3350) for HTLV-2. Among the 15 investigated communities, HTLV-1 infection occurred among the Gorotire (0.4%) and Juruna (2.0%). HTLV-2 was found to have a low prevalence (0.1–5%) among Gavião (0.4%), Aikewara Suruí (0.5%), Munduruku (0.6%), Guajajara (1.8%), and Kararaô (2.6%). A high prevalence (>10%) was observed among the Kokraimoro (11.4%), Xikrin do Bacajá (13.7%), Gorotire (22.1%) and Kubenkokre (23.1%).

### 3.2. Epidemiological Characteristics of the Population and Risk Factors Associated with HTLV-1/2 Infection

Of the 3350 Indigenous people who participated in the study, 54.3% were female. The mean age was 27.6 years (SD = 19.6), ranging from 0 to 95 years, and the most prevalent age group in the overall sample was 11 to 20 years (22.4%). This age group was also the most common among women (23.6%), while among men the predominant age group was from zero to 10 years (23.6%).

**Figure 3:**
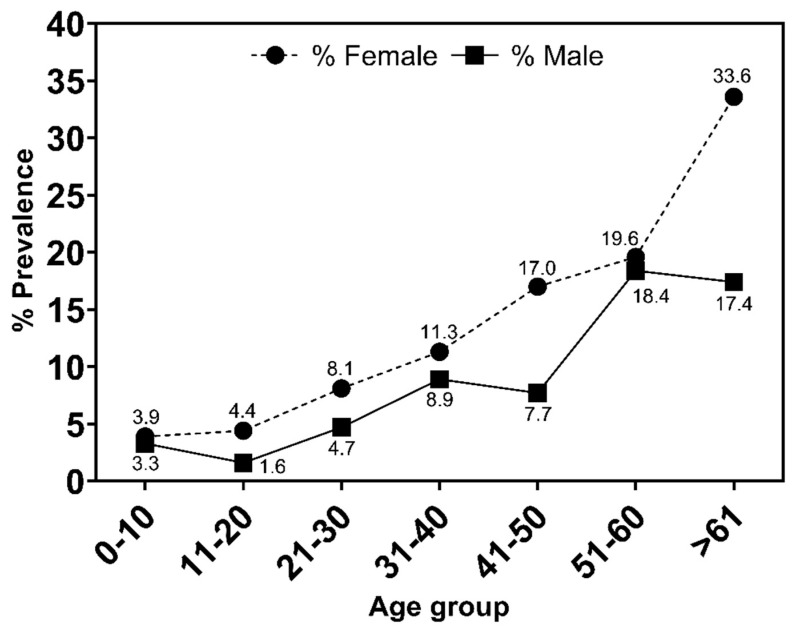
Prevalence of HTLV-1/2 infection by age and sex.

Among seropositive individuals, a higher prevalence was observed among women than men (10.1% vs. 6.5%, *p* < 0.0002). This female predominance was observed in all age groups, and in females the prevalence was significant from 41 years old (*p* < 0.0001) and in males from 51 years old (*p* < 0.0001) (Figure 3).

Of the total number of participants, 1240 individuals (56.5% female) agreed to answer an epidemiological questionnaire. Data analysis showed that 5.1% of participants reported using piercings, 5.2% reported having received blood transfusions, 88.6% said they were breastfed as children, and 79.5% reported having an active sex life, of whom 14.3% use condoms and 92.1% have only one sexual partner. When asked about the age of first sexual intercourse, 38.0% reported it being before the age of 17.

Of the epidemiological characteristics evaluated, only condom use and age at onset of sexual activity were associated with the prevalence of infection. The positivity for the infection was significantly lower among those who reported using condoms during sexual intercourse than among those who did not (1.9% vs. 86%, *p* < 0.0001). Regarding the age of onset of sexual life, positivity for infection was higher among those who started their sexual life before age 18 compared to those who started after age 18 (25.0% vs. 10.7%, *p* = 0.0204) (Table 3).

Of the three out of four HTLV-1-positive individuals, all of them stated (i) not having a piercing, (ii) not having received a blood transfusion, (iii) having been breastfed as a child and (iv) being sexually active. Two individuals stated that they had only one sexual partner and did not use condoms, while one individual did not answer the questions. When asked about the age of first sexual intercourse, one individual reported having been 17 years old, while another was 16 years old and one did not answer. Among the women (two of the four positives), one reported having seven children and having breastfed for more than 6 months, while the other reported having only two children and breastfeeding for less than 6 months.

For HTLV-2-positive women, when asked if they had already been pregnant, 87.3% answered affirmatively, of whom 35% had one to three children, 42.7% four to six children, and 21.4% reported having seven or more sons. Regarding breastfeeding, 98.3% had breastfed their children. Of these, 95.7% breastfed for six months or more. Cross-breastfeeding was reported by 14.8% of the women, only in the Kubenkokre and Kokraimoro subgroups (Table 4).

## 4. Discussion

The present study confirms the endemic infection by HTLV-2 in Indigenous peoples of the Brazilian Amazon, being hyperendemic in the Kayapó ethnic group. Our results are similar to previous findings, which showed high rates of infection among Indigenous peoples, ranging from 1.9% among the Parakanã to 57.9% among the Kayapó [6,8,9,26,27,28].

The distribution of HTLV-1/2 infection has been described as heterogeneous in the most diverse world populations [29], and these differences are attributed to factors of exposure and transmission of the virus. This heterogeneity has also been observed among Indigenous peoples. Among the 15 peoples studied in the present work, four (Araweté, Ka’apor, Tembé, Parakanã) did not present HTLV-1/2 infection, corroborating the studies by Ishak et al. and Vallinoto et al., which showed the absence of infection in the Urubu-Kaapor and Araweté peoples [6,30].

Contrary to what was observed in the present study, Ishak et al. [6] reported a 1.9% prevalence of HTLV-2 infection among the Parakanã [6]. It is possible that the only positive individual for more than 40 years was not successful in passing on the virus to other members of the community [7]. The Tembé Indigenous people were investigated for the first time, and the absence of infection among the Tembé is intriguing, considering that the contact history of this Indigenous people is ancient and clear evidence of mixing with non-Indigenous people is observed.

Among the people investigated in this study, the highest prevalence of HTLV-2 was found in subgroups of the Kayapó people, ranging from 11.4% to 23.1%. The prevalence rates described here in the Kayapó ethnic group were lower than those observed by Ishak et al. (1995), who found HTLV-2 with a prevalence of 37.4% in the Kubenkokre subgroup and 22.2% in the Kokraimoro subgroup, and by Braço et al. (2019), who showed infection in 29.0% of the individuals studied among the Xikrin do Cateté [6,8]. Much higher prevalences have also been described among the Kayapó, ranging from 22.2% to 57.9% [6,8,9,26,27,28]. These differences in prevalence may be the consequence of (i) a larger sample size for the analyses from including a larger number of people groups [31] and (ii) continued processes of fission of these peoples in recent decades, which led to the formation of new villages and the territorial dispersion of peoples and, as a consequence, the impossibility of accessing the entire population of the communities originally studied. In the current investigation, it was not possible to show serological reactivity by ELISA in the Kararaô group, contrary to what was previously known [6,12]. However, as this fact had already been reported among the Arara do Laranjal [32] people, WB tests were performed, which identified one sample as HTLV-2, one with an HTLV profile and three samples with inconclusive results. These results would have the following explanations: (i) presence of defective particles that are associated with low levels of antibodies, which are identified in asymptomatic patients and in aggressive forms of ATL [33,34]; (ii) HTLV-2 endogenization hypothesis [35]; (iii) restriction of the applicability of immunoenzymatic assays to detect antibodies to HTLV-2c, predominantly found among indigenous peoples of the Brazilian Amazon [7,36]; and (iv) possible cross-reaction with other endemic infections in the Amazon, such as malaria [37].

The Indigenous peoples who exhibited the lowest prevalence of HTLV-2 in this study were Guajajara (1.8%), Munduruku (0.6%), Aikewara-Suruí (0.5%) and Gavião (0.4%). Among these, infection has been previously described with a higher prevalence among the Munduruku (8.1%) [6]. The Guajajara, Juruna, Aikewara-Suruí and Gavião peoples had not yet been investigated for the infection. A possible association between language and infection rates was observed. For example, people from the Tupi linguistic group exhibit lower prevalence of infection, while people from the Macro-Jê language group, such as the Kayapó, exhibit a higher prevalence of the virus. On the other hand, it is important to consider that factors such as interethnic mixture, geographic isolation and sociodemographic factors related to the processes of fission or fusion of Indigenous populations can interfere, increasing or decreasing the dissemination and maintenance of the HTLV-1/2 virus among Indigenous peoples [30].

Although the occurrence of HTLV-1 infection in Amazonian Indigenous peoples is not as frequent as that observed for HTLV-2, we report here the presence of this viral type among the Juruna for the first time and Gorotire, with lower rates than previously described in the Aukre village (8.3%) by Ishak et al. [6]. The occurrence of HTLV-1 in the Juruna people can be explained by the interethnic mixture with non-Indigenous people observed in this ethnic group, evidenced by the finding that there are few individuals with Indigenous fathers and mothers in this group. Among the Gorotire people (Kayapó), it is well known, especially among men, to participate in socioeconomic activities that began in the 18th century, such as mining and logging, as well as selling handicrafts, going to bank branches and buying groceries in the nearest towns, where they often end up having sex with sex workers. The Kayapó women, in turn, have the habit of staying in the villages, overseeing the house and the children, and making handicrafts that are sold by the men [13,38].

Our results highlight the significant difference in the prevalence of HTLV infection between sexes, with women over 61 years of age being the most affected. These results corroborate previous studies indicating that women are highly affected due to more efficient transmission from men to women [8,39,40].

Mother-to-child transmission is shown to be the main form of maintenance of HTLV-2 endemicity among Indigenous peoples, commonly epidemiologically closed and semiclosed [6]. The results presented highlight the high prevalence of infection among children up to 10 years of age, showing an equal frequency between the sexes through intrauterine transmission and breastfeeding.

However, from this age group onward, sexual transmission seems to be the most important in the dissemination of the virus among Indigenous peoples. Three important epidemiological markers were shown to be associated with sexual transmission as follows: condom use, age at first sexual intercourse and number of pregnancies. It was evident that individuals who do not use condoms are more affected, since there is still a great rejection by Indigenous men of condom use, reinforcing the greater risk of transmission from men to women [6,9,12,14,27], as well as the number of children being positively correlated with the increase in the prevalence of the infection. Although the significance of prolonged breastfeeding and cross-breastfeeding has not been shown, these procedures are common in these communities and may be important forms of transmission of the virus [12,41].

## 5. Conclusions

The results presented here show the restricted occurrence of HTLV-1 infection and the high endemicity of HTLV-2 among indigenous peoples in the Brazilian Amazon, reinforcing the hyperendemic nature of the Kayapó ethnic group. The high prevalence of infection among indigenous peoples must be a reflection of different epidemiological profiles, such as sexual transmission due to not using condoms, breastfeeding, especially in cases of cross-breastfeeding, and the high rate of pregnancy in the villages. In addition, the founder effect, a phenomenon in which small groups of individuals form a new community or population and take in their composition a random sample of carriers of the virus from the original population, socio-cultural and geographic isolation, as well as the reduced sample number in some peoples, may explain the epidemiological profile in the indigenous populations examined.

## Figures and Tables

**Table 1:** Description of the people studied in the present study.

Population	Linguistic Trunk	N Sample	City of Origin/State	Geographic Coordinates
Tembé	Tupi	186	Capitão Poço/Paragominas/PA	1°50′47.7″ S 46°58′56.2″ O/ 2°17′38.5″ S 48°20′18.4″ O
Guajajara	Tupi	54	Grajaú/MA	5°21′59.076″ S 45°55′14.232″ O
Ka’apor	Tupi	21	Centro Novo/MA	3°4′1.3″ S 46°17′55.5″ O
Amanayé	Tupi	74	Paragominas/PA	3°17′51.5″ S 48°24′33.8″ O
Gavião	Macrojê	252	Bom Jesus do Tocantins/PA	5°09′21.0″ S 48°55′37.3″ O
Aikewara-Suruí	Tupi	215	São Geraldo do Araguaia/PA	5°56′00.1″ S 48°38′22.8″ O
Juruna	Juruna	100	Anapu/PA	3°30′11.8″ S 51°48′8.4″ O
Kararaô	Macrojê	39	Altamira/PA	3°54′39.9″ S 52°48′14.9″ O
Araweté	Tupi	468	Altamira/PA	4°53′16.9″ S 52°26′53.7″ O
Xikrin do Bacajá	Macrojê	401	Anapu/PA	4°37′34.5″ S 51°29′38.9″ O
Parakanã	Tupi	211	São Félix do Xingu/PA	5°39′17.1″ S 52°02′37.4″ O
Kokraimoro	Macrojê	140	São Félix do Xingu/PA	7°26′52.1″ S 52°39′33.8″ O
Gorotire	Macrojê	474	Cumaru do Norte/PA	7°46′9.5″ S 51°7′50.8″ O
Kubenkokre	Macrojê	398	Novo Progresso	8°30′0.3″ S 53°19′20.2″ O
Munduruku	Tupi	317	Jacareacanga/PA	6°26′46.8″ S 57°58′45.8″ O

**Table 2:** Prevalence of HTLV-1/2 infection in Indigenous peoples of the Brazilian Amazon.

			qPCR				Western blot					Inno-Lia					Total (%)				
People	N	EIA+	HTLV-1	HTLV-2	Neg	No	HTLV-1	HTLV-2	HTLV	Ind	Neg	HTLV-1	HTLV-2	HTLV	Ind	Neg	HTLV-1	HTLV-2	HTLV	Ind	Neg
Tembé	186	0																			
Guajajara	54	1			1								1					1 (1.8)			
Ka’apor	21	0																			
Amanayé	74	0																			
Gavião	252	1		1														1 (0.4)			
Aikewara-Suruí	215	2			2								1			1		1 (0.5)			1 (0.5)
Juruna	100	3	2		1						1						2 (2.0)				1 (1.0)
Kararaô	39	0			39			1		3	11					24		1 (2.6)		3 (7.7)	35 (89.7)
Araweté	468	0																			
Xikrin do Bacajá	401	61		45	15	2		3	2				7	1	3	1		55 (13.7)	3 (0.7)	3 (0.7)	1 (0.2)
Parakanã	211	0																			
Kokraimoro	140	18		14	3	1		2	1	1								16 (11.4)	1 (0.7)	1 (0.7)	
Gorotire	474	108	2	99	6	1		6	1								2 (0.4)	105 (22.1)	1 (0.2)		
Kubenkokre	398	93		85	6	2		7		1								92 (23.1)		1 (0.2)	
Munduruku	317	2		1		1		1										2 (0.6)			
Total	3350	289	4	245	73	7		20	4	5	12		9	1	3	26	4 (0.1)	274 (8.2)	5 (0.1)	8 (0.2)	38 (1.1)

EIA: ELISA; Neg: negative; Ind: indeterminate; No: no material for DNA extraction.

**Table 3:** Epidemiological characteristics of the study population.

Risk Factors	Positive *n* = 196 (%)	Negative *n* = 1.044 (%)	Total *n* = 1.240 (%)	*p* Values
Piercing				0.8946 ^a^
Yes	10 (5.1)	53 (5.1)	63 (5.1)	
No	183 (93.4)	955 (91.5)	1.138 (91.8)	
Uninformed	3 (1.5)	36 (3.4)	39 (3.1)	
Blood transfusion				0.1748 ^a^
Yes	6 (3.1)	59 (5.7)	65 (5.2)	
No	175 (89.3)	892 (85.4)	1.067 (86.0)	
Uninformed	15 (7.7)	93 (8.9)	108 (8.7)	
Breastfeeding as a child				0.1019 ^a^
Yes	180 (91.8)	919 (88.0)	1.099 (88.6)	
No	4 (2.0)	51 (4.9)	55 (4.4)	
Uninformed	12 (6.1)	74 (7.1)	86 (6.9)	
First sexual intercourse				0.0204 *^a^
≤17 years old	49 (25.0)	422 (40.4)	471 (38.0)	
≥18 years old	21 (10.7)	90 (8.6)	111 (9.0)	
Uninformed	126 (64.3)	532 (51.0)	658 (53.1)	
Sexually active				0.8864 ^a^
Yes	157 (80.1)	829 (79.4)	986 (79.5)	
No	32 (16.3)	178 (17.0)	210 (16.9)	
Uninformed	7 (3.6)	37 (3.5)	44 (3.5)	
Condom use	*n* = 157	*n* = 829	*n* = 986	<0.0001 *^a^
Yes	3 (1.9)	138 (16.6)	141 (14.3)	
No	135 (86.0)	591 (71.3)	726 (73.6)	
Sometimes	7 (4.5)	72 (8.7)	79 (8.0)	
Uninformed	12 (7.6)	28 (3.4)	40 (4.1)	
Number of sexual partners	*n* = 157	*n* = 829	*n* = 986	0.6541 ^b^
1	143 (91.1)	765 (92.3)	908 (92.1)	
2	2 (1.3)	5 (0.6)	7 (0.7)	
3 or more	3 (1.9)	21 (2.5)	24 (2.4)	
Uninformed	9 (5.7)	38 (4.6)	47 (4.8)	

* Significant *p* value, ^a^ Pearson’s chi-square test, ^b^ Test G; for statistical analyses, uninformed data were not considered.

**Table 4:** Epidemiological characteristics of the women studied.

Variables	% Total	% Positive
Gestational rate	85.0 (590/694)	87.3 (117/134)
Breastfeeding rate	97.3 (574/590)	98.3 (115/117)
Cross-breastfeeding rate	7.5 (43/574)	14.8 (17/115)
Number of pregnancies	*n* = 590	*n* = 117
1–3	50.0 (295)	35.0 (41)
4–6	31.4 (185)	42.7 (50)
7 or more	18.1 (107)	21.4 (25)
Uninformed	0.5 (3)	0.9 (1)
Breastfeeding time	*n* = 574	*n* = 115
Less than 6 months	2.8 (16)	2.6 (3)
6 months or more	95.5 (548)	95.7 (110)
Uninformed	1.7 (10)	1.7 (2)

## Data Availability

The data analyzed in this study are included within the paper.
